# Mapping anxiety symptoms and disordered eating using the EPSI: a latent profile analysis accounting for peak alcohol use

**DOI:** 10.1186/s40337-025-01299-7

**Published:** 2025-06-02

**Authors:** Elizabeth A. Claydon, Rose Marie Ward, Rachel B. Geyer, Donovan Weekley

**Affiliations:** 1 Department of Social and Behavioral Sciences, West Virginia University School of Public Health, Morgantown, WV USA; 2 Department of Psychology, University of Cincinnati, Cincinnati, OH USA; 3 Department of Psychology, Miami University, Oxford, OH USA; 4 Department Epidemiology & Biostatistics, West Virginia University, Morgantown, WV USA

**Keywords:** Anxiety, Distress tolerance, Anxiety-sensitivity, Disordered eating, Colleges & universities

## Abstract

This study looked at disordered eating behaviors in college students to understand the relationship between different aspects of anxiety and disordered eating. The Eating Pathology Symptoms Inventory (EPSI) questionnaire was used to help group students based on their disordered eating patterns. There were five different groups identified, although most students (54%) had lower levels of disordered eating. These groups were: 1) those who exercised too much and focused on muscle building; 2) those with less disordered eating; 3) those who were unhappy with their bodies and binged and restricted; 4) those who had moderate disordered eating and bingeing; and 5) those with high levels of disordered eating. By identifying these different groups, especially one with high anxiety and disordered eating, we can better understand these relationships in college students. This research also can assist colleges in developing more targeted services for their students.

**Supplementary Information:**

The online version contains supplementary material available at 10.1186/s40337-025-01299-7.

## Abstract (structured)

**Objective:**

Disordered eating (DE) is associated with a plethora of psychological risk factors, including anxiety and substance use disorders. The Eating Pathology Symptoms Inventory (EPSI) is a validated questionnaire to assess DE. There are no latent profile analyses (LPA) of the EPSI that also examines these profiles with regards to important psychological risk factors. The purpose of this study was to fill that gap by examining latent profiles of the EPSI subscales with respect to anxiety, distress tolerance, anxiety sensitivity, and alcohol use.

**Methods:**

The sample comprised of 1,362 college students from a midwestern university who participated in an online health survey. The EPSI scale profiles were identified using LPA with robust maximum likelihood estimation, and analyses were run to determine if the profiles differed across anxiety, anxiety sensitivity, distress tolerance, and alcohol use.

**Results:**

A five-profile solution was found to be optimal (entropy > 0.96). Profile 1 (9.7%) is defined as Excessive Exercise & Muscle Building; Profile 2 (54.1%) is a profile of Low Disordered Eating. Profile 3 (20.2%) was Body Dissatisfaction & Binge Restrict Cycle, which illustrated a potential Anorexia Binge-Purge subtype. Profile 4 (8%) was defined by Moderate Disordered Eating and Bingeing. Finally, Profile 5 (7.9%) was expressed as High Disordered Eating and was associated with high levels of anxiety and alcohol problems.

**Conclusions:**

Several latent profiles were found for the EPSI subscales and Profile 5 represented the most problematic levels. Identifying this subgroup of college students may help understand the unique presentations of DE among this population and lead future directions for how to address these intersectional student mental health concerns.

**Supplementary Information:**

The online version contains supplementary material available at 10.1186/s40337-025-01299-7.

## Introduction

Disordered eating (DE) encompasses not only clinical eating disorder behaviors, but also subclinical DE behaviors (e.g., body image disturbance; bingeing, purging, or restricting occasionally; excessive exercise, etc.), all of which are associated with negative outcomes. Among college students, the prevalence of DE behaviors has been placed between 31 and 34% based on single and larger scale studies [1, 2]. DE is also associated with many psychological factors, including anxiety, anxiety sensitivity, and distress tolerance. The purpose of this secondary data analysis is to conduct an LPA of the Eating Pathology Symptoms Inventory (EPSI) within a college population. Secondly, the goal is to understand the EPSI profiles in college students across EPSI subscales and differences in psychological risk factors (anxiety, anxiety sensitivity, and distress tolerance), while accounting for peak alcohol use.

The Eating Pathology Symptoms Inventory (EPSI [3]) assesses various facets of disordered eating in one succinct questionnaire. It has been translated and validated in a number of different languages [4, 5] and can be used in both adolescents and adults [6]. To our knowledge, current latent profile analyses (LPA) of the EPSI scales have not been examined with respect to levels of the psychological risk factors of anxiety, anxiety sensitivity, distress tolerance, while accounting for alcohol use, which are common concerns among university students. LPAs are particularly helpful because they can identify subpopulations within a larger population using a set of variables to characterize the subgroups. This process can help inform prevention, intervention and/or treatment strategies for the subpopulations that are more tailored to their specific needs. The LPAs that have been conducted with the EPSI have either focused on EDs specifically rather than DE [7], do not consider potential associations with specific psychological risk factors including the nuances of anxiety sensitivity and distress tolerance [8], have not accounted for alcohol use in college populations, are not conducted in college populations, or have been used to characterize pathological exercise rather than other DE symptoms [8]. Drawing connections between subscale profiles and risk factors is crucial for tailoring interventions among student populations who present with DE symptoms. Further, DE has a multifaceted nature with groups of unique symptoms. Thus, being able to understand distinctions in DE profiles can also highlight the differences in DE presentations and help understand if people across these profiles respond differently to interventions or treatment.

A number of psychological risk factors are currently linked with DE in college students including distress tolerance, anxiety and anxiety sensitivity. Anxiety and its facets may have shared etiological connections with DE, although temporality can be hard to untangle. Cognitive models of anxiety also help to explain the mechanisms of their association. These models suggest that anxiety, anxiety sensitivity, and distress tolerance arise when situations are perceived to be dangerous based on someone’s world view [9] Additionally, individuals may reinforce anxiety through safety behaviors and avoidance strategies, which may exacerbate their anxiety symptoms [10].

These specific areas are critical to address because they are unique facets of anxiety, which is growing most rapidly among college-aged individuals (18–25) [11]. This burgeoned during COVID with the prevalence in US university students reaching as high as 56% [12]. Women also had a higher prevalence of anxiety than men, who are concomitantly more likely to experience DE. Additional recent research has suggested that college students with anxiety and no or low levels of depression are at high risk of a suicide attempt [13]. Since DE and eating disorders are also highly associated with suicidality, this represents a vulnerable population in need of specific interventions with a transdiagnostic approach.

There are unique differences between these different facets of anxiety, which will be delineated further. Distress tolerance, whose definition is heterogenous in different applications of psychopathology research, refers to a measure of one’s ability to withstand negative stressors [14]. Distress tolerance has specifically been identified as a mediator between perfectionism and DE within a population of racially and ethnically diverse college-attending young adults [15]. Comorbidity between anxiety and EDs/DE is well established in the literature, with treatment approaches such as exposure therapy used in both conditions [16]. Anxiety refers to the negative physical and/or emotional sensations one feels in the absence of a threat or that are disproportionately severe in relation to a threat; anxiety is considered a clinical disorder when these sensations preclude one from leading a normal life [17]. In relation to anxiety, anxiety sensitivity refers to one’s own fear of an anxiety-related sensations [18, 19]. Accordingly, those college students with higher anxiety sensitivity seem prone to have maladaptive eating experiences and patterns as they attempt to cope with negative emotions through DE behaviors [20]. In that way, DE is used as a means of emotion avoidance, since these symptoms have been suggested as suppressing or distracting from negative emotions [21].

Additionally, DE and alcohol use have a strong comorbidity as shared risk behaviors, especially among a college-aged population [22]. Bulimic DE behaviors are associated with greater alcohol use and there is an indication that this shared comorbidity may be related to impulsivity [23]. Alcohol has also been noted as a coping mechanism for those participating in DE behaviors, in order to avoid or mitigate negative emotions.

Based on current theories on DE profiles and findings (i.e., studies finding three [16] to five [15] latent profiles), we posit that in a non-clinical university sample, we would find at least three latent profiles across the subscales of the EPSI. Our first aim was to characterize the latent profiles of the EPSI subscales (exploring a 3–7 profile solution). As part of the exploration of the latent profiles, peak drinking was used as a covariate, given alcohol’s strong and consistent relationship with eating disorders and DE in the literature and within this population [22, 24]. Our second aim was to connect and understand key psychological risk factors, such as distress tolerance, anxiety sensitivity, and anxiety across the latent profiles of the EPSI subscales to inform the presentation of DE across this population. There is less known about DE and distress tolerance or anxiety sensitivity, so this study aims to understand more of the nuances of those and the EPSI. This differentiation will be helpful in developing interventions for the different EPSI profiles.

## Methods

### Procedures

Undergraduate and graduate students (*N* = 17,114) enrolled full-time at a midsized midwestern U.S. university were invited to participate in an online Student Health survey. The anonymous survey was open for three weeks in March 2022; after completing, students received a $3 e-gift card for a local coffee shop. A total of 18% of those full-time students (18.07% response rate) completed the survey. In order to reduce ordered response effect (i.e., fewer participants responding to the same questionnaires that always appear at the end of the survey), we randomized survey measures, meaning some measures were only given to a subset of the full sample. The EPSI fell into this category; thus the final sample for this secondary data analysis included 1,362 participants (44.05% of those who participated). There were no significant differences across demographic variables for those who received the EPSI compared to those who did not have the EPSI presented to them in their survey. Mental health resources were provided in consent and debriefing documents to all students. This study was approved by the referent university’s Institutional Review Board (IRB#: 01191r).

### Measures

Demographics commonly associated with DE were assessed (e.g., gender identity, sexual orientation, race/ethnicity, age, fraternity/sorority status) in additional to the psychological risk factors (anxiety, anxiety sensitivity, distress tolerance, and peak drinking). Means, standard deviations, and Cronbach’s alpha estimates are provided in Table 1 for each study measure and are detailed below.

**Table 1:** Correlations, means, and standard deviations of the EPSI with psychological risk factors

	EPSI									
	Body Dissatisfaction	Binge Eating	Excessive Exercise	Cognitive Restraint	Purging	Neg. Att. Towards Obs	Restricting	Muscle Building	Alpha	M (SD)
GAD-7	0.42***	0.26***	0.05	0.24***	0.15***	0.05	0.37***	0.06*	0.93	6.71 (5.89)
DTS Tolerance	− 0.24***	− 0.15***	0.05	− 0.08**	− 0.10***	− 0.02	− 0.21***	0.03	0.80	3.28 (1.01)
DTS Absorption	− 0.32***	− 0.21***	0.05	− 0.11***	− 0.10***	0.00	− 0.24***	0.01	0.85	3.09 (1.10)
DTS Appraisal	− 0.28***	− 0.22***	0.03	− 0.12***	− 0.17***	− 0.04	− 0.24***	− 0.03	0.85	3.38 (0.87)
DTS Regulation	− 0.20***	− 0.15***	− 0.05	− 0.11***	− 0.10***	− 0.04	− 0.19***	− 0.03	0.81	3.25 (0.98)
ASI Physical Concerns	0.31***	0.27***	0.04	0.18***	0.22***	0.09***	0.31***	0.08**	0.90	5.80 (5.76)
ASI Cognitive Concerns	0.34***	0.32***	0.07*	0.24***	0.27***	0.13***	0.32***	0.11***	0.92	5.98 (6.13)
ASI Social Concerns	0.40***	0.29***	0.07**	0.25***	0.17***	0.13***	0.31***	0.07**	0.84	9.54 (6.08)
Peak Drinking	− 0.04	0.05*	0.20***	0.04	0.01	0.18***	− 0.01	0.23***	--	4.29 (4.50)
Alpha	0.89	0.86	0.88	0.77	0.91	0.91	0.89	0.82		
M (SD)	10.74 (7.21)	6.19 (5.04)	5.53 (5.19)	3.98 (3.07)	1.71 (3.72)	3.91 (4.54)	5.73 (5.52)	3.35 (4.11)		

Note. M (SD) = Mean (Standard Deviation). * *p* <.05; ** *p* <.01; *** *p* <.001. GAD7 = Generalized Anxiety Disorder– 7. DTS = Distress Tolerance Scale. ASI = Anxiety Sensitivity Index. Peak drinking = the highest number of standard drinks in the past 30 days. Neg. Att. Towards Obs = Negative Attitudes towards Obesity

#### Disordered eating symptomatology measured by the, *eating pathology symptom inventory (EPSI)*

The *EPSI* assessed DE symptoms and consists of a total of 45 Likert scale items (ranging from *Never* to *Very Often*) with eight separate subscales: Body Dissatisfaction (e.g., “I did not like how clothes fit the shape of my body”), Binge Eating (e.g., “I stuffed myself with food to the point of feeling sick”), Cognitive Restraint (e.g., “I counted the calories of foods I ate”), Purging (e.g., “I used diuretics in order to lose weight”), Restricting (e.g., “I skipped two meals in a row”), Excessive Exercise (e.g. “I pushed myself extremely hard when I exercised”), Negative Attitudes towards Obesity (e.g., “I felt that overweight people are unattractive”), and Muscle Building (e.g., “I used protein supplements”) [3]. The EPSI displays excellent estimates of validity, internal consistency (α = 0.84–0.89) and test-retest reliability (Pearson *r* =.73).^3^ Within our sample, we found high internal reliability (α = 0.77 to 0.91) across the subscales.

#### Anxiety measured by the *general anxiety disorder-7 (GAD-7)*

The GAD-7 is a seven-item measure with a 4-point Likert scale that assesses level of anxiety over the last two weeks [25] Item answers range from *Not at all* to *Nearly Every Day* and are scored from 0 to 3; total scores range from 0 to 21. A cut point of 10 indicates a potential case of generalized anxiety disorder and the scale has good sensitivity (89%) and specificity (82%) at that cut point (Spitzer et al., 2006). For purposes of these analyses, we used it as a continuous measure. Example items include “Not being able to stop or control worrying” and “being so restless that it is hard to sit still.” Internal consistency was 0.93 in our sample.

#### Distress tolerance measured by the *distress tolerance scale*

The Distress Tolerance Scale [26] consists of 15, 5-point Likert-scale items to detect an individual’s self-reported ability to handle unpleasant emotional states. Items are answered from *Strongly Disagree* to *Strongly Agree* with example questions such as: “There’s nothing worse than feeling distressed or upset,” and “My feelings of distress or being upset are not acceptable.” The scale covers four different types of distress, such as perceived ability to tolerate emotional distress (Tolerance), subjective appraisal of distress (Appraisal), absorption by negative emotions (Absorption), and regulation efforts to alleviate distress (Regulation). Higher levels of DTS indicate better distress tolerance. Internal consistency ranged between 0.80 and 0.85 for the DTS subscales.

#### Anxiety sensitivity measured by the *anxiety sensitivity index-3*

The ASI-3 is an 18-item self-report questionnaire that identifies concerns that someone has around their anxiety symptoms [19]. There is a 5-point Likert scale for answers which ranges from *Very Little* to *Very Much*, and points are allocated from 0 to 4. The ASI-3 scoring includes a total from the summed scores, as well as 3 subscales: Physical Concerns (e.g., When I feel pain in my chest, I worry that I’m going to have a heart attack), Social Concerns (e.g., “I worry that other people will notice my anxiety”), and Cognitive Concerns (e.g., “When my mind goes blank, I worry there is something terribly wrong with me”). Higher scores indicate greater anxiety sensitivity. Internal consistency ranged between 0.84 and 0.90 for the ASI subscales.

#### Alcohol consumption indicated by *peak drinking*

Participants were given the definition of a standard drink of alcohol (i.e., one 12-ounce beer, one 1.5 ounce shot of liquor, or a five-ounce glass of wine). Peak drinking was assessed with the following question: “During the last 30 days, what is the highest number of drinks that you drank on any one occasion?” Participants were also asked how many drinks they have on a typical day when drinking.

### Data analysis

To explore potential subgroups, profiles or underlying groups from the EPSI scales were identified using LPA in Mplus 8.8 with robust maximum likelihood estimation. LPA is a person-centered approach (vs. a variable centered approach) and uses latent variable mixture modeling with a categorical latent variable and continuous manifest variables. LPA uses a stepwise approach to determine the optimal solution. In LPA, it is assumed that the optimal model (1) specifies the number of profiles, (2) each participant only belongs in one profile, and (3) participants within a profile are homogenous.

The first phase is to define the population and selected the indicators. For this study, we examined a college student population and the EPSI subscales were chosen to develop the profiles because it reduced the number of parameters to be estimated and provided a better approximation for the continuous indicators.

In the second phase, the researchers tested modeling assumptions and examined the patterns of missing data. The Purging subscale had the most extreme values. These values were retained and untransformed since they were plausible values for the scale. Patterns of missing variables were examined using Little’s MCAR test. It determined that the data was missing completely at random, Little’s MCAR, χ^2^(89) = 86.20, *p* =.56. In Mplus, Full Information Maximum Likelihood (FIML) was used to handle the missing data. A power analysis based on the recommendations of Tein et al. 27] suggests that the current study design has sufficient sample size to detect small effect sizes for a LPA using the 8 EPSI subscales for 4, 5, and 6 profile solutions. Given that Forbush and Wildes (2017) [7] presented a 5-profile solution for the EPSI using a clinical sample of participants with EDs and the results of our power analysis, we determined that we had a sufficient sample size for our design.

Next, the third phase was comprised of model estimation (i.e., profile enumeration and model formulation). We estimated 1 to 7 profiles. After estimating a series of profiles with this model (also known as the profile-invariant diagonal model) and determining the model of best fit for the default model, we systematically tested profiles varying the constraints.

In the next phase, the models were evaluated based on fit and classification diagnostics. The number of iterations was set at 500. To further determine the optimal solution, the Akaike Information Criteria (AIC) and Bayesian Information Criteria (BIC) were used (lower values indicate better model fit [28]). Moreover, the Lo-Mendell-Rubin likelihood ratio test (LMR), an assessment of relative fit, examined the null hypothesis that the *k*-1 class solution is acceptable compared to the *k*-class solution (*p* <.05 suggests that the *k-*class model has preferable fit [29]). There is no single best fit index recommended in the literature. Therefore, multiple indices were used to determine the optimal solution. In addition to the evaluation of fit, we reviewed the classification diagnostics. Entropy was used to examine classification accuracy or separation (higher values indicate better classification/separation; 0.80 is considered high entropy). In addition, we also examined the models with the number of groups that had less than 5% of cases or less than 50 people. Finally, we examined the average latent class posterior probability or a measure of classification accuracy. Values greater than 0.70 are preferred [30]. Average latent class posterior probability are presented in a matrix with the diagonal representing the average probability that a person is assigned to a class given their scores on the indicator variables used to estimate the profiles. Scores closer to 1.0 on the diagonal are desirable. Off-diagonal values are the probability of the cases that belong in the other profiles in the current solution. Scores closer to 0 are desirable and indicate low probability. A cut off of 0.90 is desirable for the diagonal values [31].

Then, the researchers determine if distal outcomes or covariates were needed. After the optimal number of latent profiles was determined, we added peak drinking as a covariate and distal outcomes (GAD-7, DTS, and ASI-3 subscales) to the models to determine if including them as additional profile indicators would impact the solution [32] By adding covariates (R3STEP method) and distal outcomes (BCH method), we were able to further describe the profiles and analyze the validity of the solution. The R3STEP method has three steps: (1) Conduct the LPA to identify latent profiles without including covariates; (2) Assign individuals to latent profiles based on their posterior probabilities from the LPA. This step involves creating a most likely class membership variable; and (3) (R3STEP) Use the covariates to predict latent profile membership. This is done by modeling the relationship between the covariates and the most likely class membership variable, typically using multinomial logistic regression. We used the BCH method for including the distal outcomes [33]. The BCH method is a technique used to handle auxiliary variables after the latent profiles have been identified. BCH stands for Bolck, Croon, and Hagenaars, the researchers who developed this method. It incorporates auxiliary variables into the analysis without affecting the estimation of the latent profiles.

Finally, the profiles were examined and compared with respect to other variables not used in the model building phase (e.g., demographic variables and outcome variables). Chi-square tests of independence examined the profiles across the demographic categories. Follow up pairwise Wald tests examined differences among the profiles across the outcome variables.

## Results

### Participants

A majority of participants (67.1%, *n* = 914) identified as a woman; identified predominantly as heterosexual (74.7%, *n* = 1018), and were White (88.2%, *n =* 1201), There was a slight over sampling of first year students (36.5%, *n* = 497) and the average age was 20.44 (*SD* = 3.16) years. Approximately 31.1% (*n* = 424) indicated that they were members of a social sorority or fraternity. Most participant characteristics are similar to those of the student population from which it was sampled, except for a slight oversampling of women and first year students. See Table 2 for full demographics.

**Table 2:** Demographic table

	Percent	*n*
**Gender Identity**		
Woman	67.2	914
Man	29.3	399
Non-binary	2.9	40
Prefer not to answer	0.6	8
**Sexual Orientation**		
Heterosexual	74.9	1018
Bisexual	11.9	162
Queer	3.2	43
Lesbian	2.5	34
Questioning	1.8	25
Asexual	1.5	20
Gay	1.4	19
Other	2.8	38
**Race/Ethnicity**		
White	88.2	1201
Asian or Asian Am.	10.0	136
Hispanic or Latina/o	4.0	54
Black or African Am.	3.5	48
Native Am. or Alaskan	0.7	9
Hawaiian or PI	0.5	7
Other	1.8	24
**Year in School**		
First Year	36.5	497
Second Year	23.9	325
Third Year	17.2	234
Fourth Year	12.2	166
Fifth Year	1.2	17
Graduate Students	7.0	96
**International Students = Yes**	12.5	170
**Sorority or Fraternity = Yes**	31.1	424
**Living Situation**		
On-campus	60.3	761
Off campus with friends or alone	30.4	384
Off-campus with parents	4.1	52
Off-campus with spouse/partner and/or children	3.8	48
Fraternity House	1.4	18
**The average age was 20.44 (*SD*** **= 3.16) years**		

### Profiles of the EPSI scales

Fit statistics for the unconditional 1–6 LPA Models for the EPSI scales are presented in Supplementary Table 1. The fit criteria or information indexes (e.g., BIC) decrease as the number of profiles is tested. The 3-, 4-, 5-, and 6-profile solutions resulted in the smallest profile representing around 7–8% of the cases. Based on interpretability, the LMR likelihood ratio test, a 5-profile solution was determined to be optimal (entropy > 0.82) from the unconditional model. Estimates for the 5-profile model was examined twice with different seeds and determined to be stable. The average latent class posterior probability indicated good profile separation. See Supplementary Table 2 for more information.

**Fig. 1:**
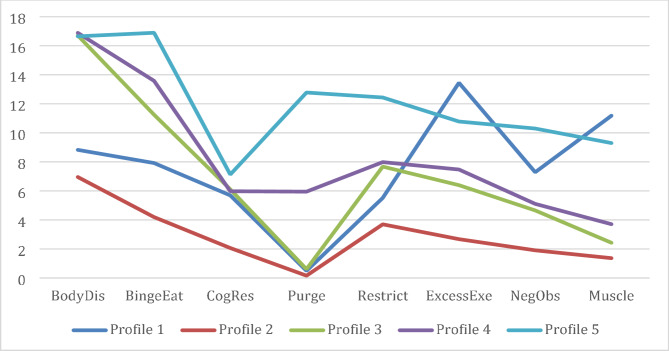
Visual representation of the 5-profile solution of latent profiles with mean levels of the EPSI subscales. *Note.* BodyDis = Body Dissatisfaction. BingeEat = Binge Eating. CogRes = Cognitive Restraint. Purge = Purging. Restrict = Restricting. ExcessExe = Excessive Exercise. NegObs = Negative Attitudes towards Obesity. Muscle = Muscle Building

One covariate was added to the final 5-profile solution, and the results are reported in Table 3. Figure 1; Table 4 presents average scores for each class across the indicator variables used to develop the classes. Profile 1 (which will be referred to as Excessive Exercise & Muscle Building) is depicted by high levels of excessive exercise and muscle building. It comprised 9.7% (*n* = 132) of the sample. Profile 2 (Low Disordered Eating) comprised 54.1% (*n* = 734) of the sample and by the lowest levels of DE tendencies in the model. Profile 3 (Body Dissatisfaction & Binge Restrict Cycle; 20.2%, *n* = 274) was represented high levels of body dissatisfaction, moderate levels of binge eating, and moderate levels of restricting. Profile 4 (Moderate DE & Bingeing) comprised 8.0% (*n* = 109) of the sample and had the highest levels of body dissatisfaction, high levels of binge eating, and moderate levels of the other scales. Profile 5 (High DE) was the smallest class with 7.9% (*n* = 107) of the sample and for the most part had the highest level on each subscale with the exception of execessive exercise and muscle building.

**Table 3:** Descriptive statistics of indicator variables for 5-class latent profile model

	1		2		3		4		5	
**EPSI Scale**	**M**	**SE**	**M**	**SE**	**M**	**SE**	**M**	**SE**	**M**	**SE**
BodyDis	8.825	0.648	6.953	0.366	16.704	0.68	16.885	0.694	16.646	0.514
BingeEat	7.918	0.567	4.194	0.231	11.248	0.638	13.58	0.808	16.892	0.719
CogRes	5.69	0.31	2.064	0.103	6.106	0.383	5.975	0.320	7.126	0.311
Purge	0.492	0.131	0.159	0.025	0.606	0.087	5.947	0.428	12.775	0.472
Restrict	5.516	0.521	3.696	0.244	7.672	0.461	7.981	0.628	12.434	0.392
ExcessExe	13.447	0.476	2.674	0.175	6.393	0.533	7.471	0.508	10.778	0.540
NegObs	7.285	0.614	1.905	0.143	4.653	0.392	5.105	0.486	10.295	0.554
Muscle	11.178	0.574	1.365	0.088	2.426	0.173	3.706	0.396	9.295	0.577

Note. Means depicted with standard errors in parentheses. BodyDis = Body Dissatisfaction. BingeEat = Binge Eating. CogRes = Cognitive Restraint. Purge = Purging. Restrict = Restricting. ExcessExe = Excessive Exercise. NegObs = Negative Attitudes towards Obesity. Muscle = Muscle Building

**Table 4:** Multinomial logistic regression comparing EPSI profiles by covariate (3-Step Procedure)

Profile	Logit	SE	*p*	OR	95% CI
Ref = 1					
2	− 0.135	0.023	< 0.001	0.873	0.835-0.914
3	− 0.105	0.023	< 0.001	0.900	0.861-0.941
4	− 0.137	0.029	< 0.001	0.872	0.824-0.923
5	− 0.094	0.030	0.001	0.910	0.859-0.964
Ref = 2					
1	0.135	0.023	< 0.001	1.145	1.094–1.198
3	0.030	0.019	0.113	1.031	0.993 − 1.070
4	− 0.002	0.025	0.946	0.998	0.951-1.048
5	0.041	0.026	0.117	1.042	0.990-1.097
Ref = 3					
1	0.105	0.023	< 0.001	1.111	1.063–1.161
2	− 0.030	0.019	0.113	0.970	0.935-1.007
4	− 0.032	0.027	0.232	0.969	0.919-1.021
5	0.011	0.028	0.694	1.011	0.958-1.067
Ref = 4					
1	0.137	0.029	< 0.001	1.147	1.084–1.214
2	0.002	0.025	0.946	1.002	0.954-1.051
3	0.032	0.027	0.232	1.032	0.980-1.088
5	0.043	0.033	0.197	1.044	0.978-1.114

Note. Covariate = peaking drinking. Peak drinking = the highest number of standard drinks in the past 30 days. SE = standard error. Ref = reference class

### Demographics across profiles

The proportion of people who identify as men or women varied across the profiles, χ^2^(*n* = 1307, 4) = 130.265, *p* <.001. Excessive Exercise & Muscle Building (Profile 1) had the highest proportion of men with 68.7% of people in the profile identifying as men. Moderate DE & Bingeing (Profile 4) had the highest proportion of women with 87.4% identifying as women. The Low DE (Profile 2), Body Dissatisfaction & Binge Restrict Cycle Profile (Profile 3), and High DE (Profile 5) Profiles had a high proportion of women, with 68.9%, 83.2%, and 71.4% respectively. Race and Ethnicity did not significantly vary across the profiles, χ^2^(*n* = 1356, 28) = 30.696, *p* =.331. Sexual orientation also did not significantly vary across the classes, χ^2^(*n* = 1353, 32) = 38.216, *p* =.208; however, pairwise comparisons indicate that non-heterosexual individuals were also more likely to be in the Body Dissatisfaction & Binge Restrict Cycle Profile than any other. The Low DE Profile also contained more individuals in sororities or fraternities (1/3rd of the profile).

### Psychological risk factors

We used the BCH procedure in Mplus to compare the profiles across the continuous outcome variables (i.e., the psychological risk factors). The BCH process uses Wald tests to compare the mean scores of the variables across the profiles. See Supplmentary Table 3 for more information. In general, Profiles 1 and 2 tended to have the lowest levels across the GAD7, ASI scales, and distress tolerance. Profile 5 tended to have the highest levels across all of the psychological risk factor variables.

## Discussion

This study contributes to understanding the intersectionality and unique presentation of DE symptoms, especially among non-clinical samples. Individuals with DE do not experience those symptoms in isolation. Instead, individuals are likely to have other psychological risk factors that also intersect with DE symptoms. Understanding these heterogeneous presentations of DE with different anxiety symptoms is critical to identifying the broad spectrum of individuals with DE in college and creating transdiagnostic interventions for them. Of note, the subgroup with the most elevated ED symptoms also had high levels of anxiety, anxiety sensitivity, and lower distress tolerance. More research is needed on whether individuals in this profile or others may have different outcomes from treatment or interventions. Additionally, it also indicates that we should be assessing college students for both DE behaviors and anxiety symptoms rather than just one in isolation.

### Profile size

The largest profile was the Low DE profile (54.1%), illustrating that the majority of these participants had relatively low DE. However, compared with other estimates placing DE in college students at 31–34%^1–2^, the remaining profiles in our study indicate 45.8% of students showing signs of DE. However, those prior studies did not use the EPSI to evaluate DE, but rather the EDE-Q or the SCOFF, neither of which assess for excessive exercise or muscle building. This could account for some of the differences in prevalence. A longitudinal study from 2013 to 2021 also showed a substantial increase in ED risk (measured by the SCOFF) and indicated that the risk increased by 3% points during the COVID-19 pandemic [34]. Our data were collected in 2022, making it possible that this discrepancy might also be realated to increases during the pandemic.

### Profiles & disordered eating

The purpose of this study was to map psychological risk factors with a LPA of the EPSI subscales among university-attending young adults. A total of five profiles were identified using an LPA of the eight EPSI subscales, each with different patterns of DE symptomatology. Excessive Exercise and Muscle Building (Profile 1) indicated moderate body dissatisfaction as well as larger spikes in excessive exercise and muscle building, higher negative observations of obesity, and a higher proportion of university students who identified as men. This is consistent with norms for the EPSI that show men score significantly higher than women in the excessive exercise, muscle building, and negative attitudes towards obesity subscales [35]. This profile could indicate a problematic relationship with exercise where it is used as a compensatory behavior for food intake or a way of severe restricting.

### Psychological risk factors

#### Anxiety

As expected, we also saw differences between the profiles in the specific psychological risk factors of interest. The Body Dissatisfaction & Binge Restrict Cycle Profile (Profile 3) showed the highest levels of anxiety (measured by the GAD-7). This is not surprising since there is recent research to suggest that anxiety (specifically general anxiety and anxiety about getting fat) is highly associated with more binge eating symptoms [36]. There is extensive research highlighting the comorbidity of anxiety and EDs, but less that looks at this in non-clinical populations and with other psychological factors that may influence the experience of anxiety [16].

#### Anxiety sensitivity

Both anxiety sensitivity physical and cognitive concerns were highest in the High DE Profile. This is consistent with research that indicates a strong association with DE and EDs, especially since higher concerns have been associated with a higher number of DE symptoms [37]. However, anxiety sensitivity social concerns were highest among the Body Dissatisfaction and Binge Restrict Cycle Profile. Recent research has indicated that the social concerns subscale is associated with fewer symptoms, so it may be that specific symptoms such as body dissatisfaction, bingeing, and restricting may be associated with social concerns out of fear about how those symptoms would be perceived [37].

#### Distress tolerance

Poorer distress tolerance (indicated by lower DTS scores in the Tolerance subscale), absorption (DTS Absorption subscale), and regulation (DTS Regulation subscale) were found in the Body Dissatisfaction & Binge Restrict Cycle Profile as well as the Moderate DE & Bingeing Profile. Both of these profiles had high levels of bingeing, so this association with poor distress tolerance could be due to individuals using bingeing as a negative coping strategy. Since their absorption of negative emotions was poorer as well as their regulation, this indicates less ability to prevent internalizing negative feelings and cope with them in positive ways. This is highly connected to the cognitive models of anxiety, with symptom avoidance through safety behaviors. However, distress appraisal was lowest in the High DE Profile, which saw high levels of all DE behavior. This could be because these high levels of DE made students feel more distress.

#### Peak drinking

The Excessive Exercise Profile was associated with high levels of peak drinking. This profile potentially reflects the phenomenon called food and alcohol disturbance, which refers to DE patterns or compensatory behaviors (such as excessive exercise) to either increase the speed at which individuals get drunk or offset al.cohol calories [38]. The High DE Profile also was associated with higher peak drinking compared to the Low DE, Body Dissatisfaction & Binge Restrict Cycle, and Low DE Profiles.

The lowest levels of peak drinking were associated with the Low DE Profile. Additionally, over one-third of them were in sororities or fraternities, which historically has shown higher rates of DE as well as alcohol use, in contradiction to the current findings [39]. However, recent research is not showing an elevated risk for DE specifically among sororities and fraternities [40] Many DE interventions have focused on this population, which may help explain this. Additionally, alcohol use may be slightly reduced among this population due to the increased oversight of sororities and fraternities.

### Profile synthesis

When mapped against psychological risk factors, Profile 5, although a small proportion of the overall sample, showed some of the highest DE patterns as well as relatively high anxiety and the highest levels of AS (physical and cognitive subscales; and second highest social subscale), and higher peak drinking compared to Profile 2, 3, and 4. Additionally, this profile had lower levels of distress tolerance. This profile, although smaller than the others, will warrant unique interventions that can address distress tolerance and DE. This profile warrants additional research.

### Strengths & limitations

This study has many strengths including a moderately large sample size that is representative of the broader university population with a randomized survey design. Well-validated measures, such as the EPSI, GAD-7, etc. were incorporated into the survey to improve internal validity of the survey. Additionally, the presence of five profiles of DE with the EPSI aligns with the DSM approach to DE as well as Forbush et al.’s model of the need for at least three or more profiles [7, 41]. This indicates these findings in a non-clinical population are in line with theoretical and clinical understanding of DE.

However, some limitations should be noted to contextualize this research. First, the study utilized self-report data which are susceptible to biases. Second, due to the cross-sectional study design, this limits our ability to determine temporal relationships or causality. Third, although gender composition differed across the profiles, we did not include gender identity as a covariate. We did not have large enough samples for all gender identities and would have lost data when using it as a covariate. Future studies with greater gender diversity should consider gender as a covariate. Fourth, due to the length of the survey, we allowed for planned missingness by only providing certain scales to a random subset of participants but not to others. However, we still had sufficient sample for the power needed for these analyses. An additional limitation is that the EPSI does not measure the difference between objective and subjective binge episodes, so the subscale for binge eating may be either or both. The EPSI is also limited in that it does not provide a clinical cutoff to determine clinically significant pathology. However, the High DE Profile was similar and at times higher than EPSI norms for individuals with eating pathology [6]. Our sample was also limited in diversity with almost 90% of the sample, identifying as white. Finally, some of the psychological correlates, such as anxiety and distress may be manifesting as DE symptoms, since these are often co-occurring conditions and are hallmarks of some DE. However, since this is cross-sectional data, we are unable to fully tease out that relationship.

## Conclusions & implications for future research

The distinct subgroups identified by the LPA of the EPSI illustrate different DE patterns and the ways in which they are connected with psychological correlates. Understanding these different subgroups within a population of university-attending young adults can help us get a clearer view of different presentations of DE in college students. In recent years, more DE interventions have been implemented on college campuses, such as an internet-based one called Healthy Body Image [42]. This intervention screens individuals and offers them evidence-based programs based on their ED risk. Having an intervention such as this be more widespread and take into account interrelated behavior and the holistic and intersectional (e.g., anxiety, anxiety sensitivity, distress tolerance, alcohol use) nature of those they affect may help identify other areas of concern for these individuals, so they can get the help they need across symptomologies.

Furthermore, even though LPA has provided promising insight into the subgroups of university-attending young adults based on levels of DE pathology, it fails to identify any temporal relationships between the various psychological correlates. Whereas it is important to focus on the patterns that demarcate various subgroups at present, it is equally important to identify potential cause-and-effect relationships that may have developed earlier in age. It is possible that one psychological risk factor predates others and significantly increases susceptibility to the other variables measured. This said, future research should be longitudinal in nature, such that different psychological risk factors can be measured across time. This type of research can also help to tease out whether the psychological risk factors are features of DE or just co-occurring. This research may provide insight into developing evidence-based, preventative interventions against DE pathology before its onset.

## Electronic supplementary material

Below is the link to the electronic supplementary material.

Supplementary Material 1

## Data Availability

Data is available upon reasonable request.
