# Supplementary material for: Mapping anxiety symptoms and disordered eating using the EPSI: a latent profile analysis accounting for peak alcohol use
Source: J Eat Disord. 2025 Jun 2;13:96. doi: 10.1186/s40337-025-01299-7 (PMC12128327; doi:10.1186/s40337-025-01299-7)
Supplement: Supplementary file 1 — Supplementary Material 1 [file 40337_2025_1299_MOESM1_ESM.docx]

Supplementary Table 1. LPA models of the EPSI subscales

| Model  tested | -2LL | np | AIC | BIC | SSA-BIC | p-value | Entropy | % profile |
| --- | --- | --- | --- | --- | --- | --- | --- | --- |
| **Profile Invariant Diagonal** | | |  |  |  |  |  |  |
| 1 | -32452.475 | 16 | 64936.951 | 65020.347 | 64969.522 | - | - | - |
| 2 | -31153.855 | 25 | 62488.017 | 62488.017 | 62408.602 | <.00001 | .881 | 29.2 |
| 3 | -30449.032 | 34 | 60966.064 | 61143.282 | 61035.279 | 0.0003 | .886 | 8.7 |
| 4 | -30017.789 | 43 | 60121.577 | 60345.706 | 60209.113 | <.00001 | .902 | 8.2 |
| **5** | **-29637.478** | **52** | **59378.957** | **59649.996** | **59484.814** | **0.0578** | **.824** | **8.5** |
| 6 | -29159.260 | 61 | 58440.520 | 58758.470 | 58564.699 | 0.0327 | .915 | 7.0 |
| **Profile Varying Diagonal** | | |  |  |  |  |  |  |
| 1 | -32452.475 | 16 | 64936.951 | 65020.347 | 64969.522 | - | - | - |
| 2 | DNC |  |  |  |  |  |  |  |
| **Profile Invariant Unrestricted** | | | |  |  |  |  |  |
| 1 | -31193.601 | 37 | 62461.203 | 62654.058 | 62536.524 | - | - | - |
| **2** | **-29185.928** | **81** | **58533.855** | **58956.051** | **58698.748** | **<.00001** | **.898** | **30.6** |
| 3 | -29039.873 | 118 | 58315.746 | 58930.797 | 58555.961 | .1636 | .902 | 13.4 |
| **Profile Varying Unrestricted** | | | |  |  |  |  |  |
| 1 | -31193.601 | 37 | 62461.203 | 62654.058 | 62536.524 | - | - | - |
| **2** | **-28974.695** | **89** | **58127.390** | **58591.284** | **58308.568** | **<.00001** | **.910** | **28.3** |
| 3 | NR |  |  |  |  |  |  |  |

Note. -2LL = -2 log likelihood; np = number of parameters; AIC = Akaike information criteria; BIC = Bayesian information criteria; SSA-BIC = sample adjusted BIC; LMR LRT = Lo-Mendell-Rubin likelihood ratio test. DNC = did not converge.NR = solution not trustworthy due to local maxima and solution not replicated. In addition, one of the profiles had zero cases.

Supplementary Table 2. Average Latent Profile Posterior Probabilty

| Profiles | 1 | 2 | 3 | 4 | 5 |
| --- | --- | --- | --- | --- | --- |
| 1 | **.876** | .000 | .121 | .003 | .000 |
| 2 | .000 | **.891** | .092 | .012 | .005 |
| 3 | .115 | .040 | **.834** | .010 | .000 |
| 4 | .009 | .018 | .037 | **.935** | .001 |
| 5 | .000 | .013 | .001 | .007 | **.978** |

Note. Average latent profile posterior probability are presented in a matrix with the diagonal representing the average probability that a person is assigned to a Profile given their scores on the indicator variables used to estimate the profiles. Average latent Profile probabilities for most likely latent Profile membership (Row) by latent Profile (Column).

Supplementary Table 3. Equality Tests of Means Across Profiles using the BCH Procedure

| **GAD 7** | **M** | **SE** |  | **M** | **SE** |
| --- | --- | --- | --- | --- | --- |
| Profile 1 | 6.235 | .540 | Profile 2 | 5.186 | .205 |
| Profile 3 | 9.311 | .443 | Profile 4 | 9.326 | .627 |
| Profile 5 | 7.955 | .695 |  |  |  |
|  | **Chi-square** | **p-value** |  | **Chi-square** | **p-value** |
| **Overall Test** | 98.065 | <.001 | Profile 1 v. 2 | 3.277 | .070 |
| Profile 1 v. 3 | 18.417 | <.001 | Profile 1 v. 4 | 13.794 | <.001 |
| Profile 1 v. 5 | 3.795 | .051 | Profile 2 v. 3 | 63.767 | <.001 |
| Profile 2 v. 4 | 39.442 | <.001 | Profile 2 v. 5 | 14.605 | <.001 |
| Profile 3 v. 4 | .000 | .985 | Profile 3 v. 5 | 2.703 | .100 |
| Profile 4 v. 5 | 2.071 | .150 |  |  |  |
| **DTS Tolerance** | **M** | **SE** |  | **M** | **SE** |
| Profile 1 | 3.591 | .088 | Profile 2 | 3.428 | .041 |
| Profile 3 | 2.979 | .068 | Profile 4 | 2.937 | .096 |
| Profile 5 | 3.028 | .101 |  |  |  |
|  | **Chi-square** | **p-value** |  | **Chi-square** | **p-value** |
| **Overall Test** | 60.767 | <.001 | Profile 1 v. 2 | 2.839 | .092 |
| Profile 1 v. 3 | 28.934 | <.001 | Profile 1 v. 4 | 25.135 | <.001 |
| Profile 1 v. 5 | 17.716 | <.001 | Profile 2 v. 3 | 28.285 | <.001 |
| Profile 2 v. 4 | 22.331 | <.001 | Profile 2 v. 5 | 13.591 | <.001 |
| Profile 3 v. 4 | .124 | .725 | Profile 3 v. 5 | .161 | .689 |
| Profile 4 v. 5 | .414 | .520 |  |  |  |
| **DTS Absorption** | **M** | **SE** |  | **M** | **SE** |
| Profile 1 | 3.390 | .089 | Profile 2 | 3.299 | .045 |
| Profile 3 | 2.635 | .074 | Profile 4 | 2.717 | .103 |
| Profile 5 | 2.893 | .102 |  |  |  |
|  | **Chi-square** | **p-value** |  | **Chi-square** | **p-value** |
| **Overall Test** | 81.136 | <.001 | Profile 1 v. 2 | .829 | .363 |
| Profile 1 v. 3 | 40.406 | <.001 | Profile 1 v. 4 | 24.019 | <.001 |
| Profile 1 v. 5 | 13.387 | <.001 | Profile 2 v. 3 | 52.400 | <.001 |
| Profile 2 v. 4 | 26.769 | <.001 | Profile 2 v. 5 | 13.335 | <.001 |
| Profile 3 v. 4 | .406 | .524 | Profile 3 v. 5 | 4.213 | .100 |
| Clsas 4 v. 5 | 1.416 | .234 |  |  |  |
| **DTS Appraisal** | **M** | **SE** |  | **M** | **SE** |
| Profile 1 | 3.595 | .067 | Profile 2 | 3.560 | .034 |
| Profile 3 | 3.105 | .062 | Profile 4 | 3.085 | .087 |
| Profile 5 | 2.984 | .076 |  |  |  |
|  | **Chi-square** | **p-value** |  | **Chi-square** | **p-value** |
| **Overall Test** | 91.152 | <.001 | Profile 1 v. 2 | .221 | .639 |
| Profile 1 v. 3 | 27.159 | <.001 | Profile 1 v. 4 | 21.385 | <.001 |
| Profile 1 v. 5 | 35.888 | <.001 | Profile 2 v. 3 | 36.326 | <.001 |
| Profile 2 v. 4 | 25.920 | <.001 | Profile 2 v. 5 | 47.316 | <.001 |
| Profile 3 v. 4 | .034 | .853 | Profile 3 v. 5 | 1.513 | .219 |
| Profile 4 v. 5 | .734 | .392 |  |  |  |
| **DTS Regulation** | **M** | **SE** |  | **M** | **SE** |
| Profile 1 | 3.338 | .086 | Profile 2 | 3.422 | .040 |
| Profile 3 | 2.963 | .064 | Profile 4 | 2.968 | .092 |
| Profile 5 | 3.039 |  |  |  |  |
|  | **Chi-square** | **p-value** |  | **Chi-square** | **p-value** |
| **Overall Test** | 50.552 | <.001 | Profile 1 v. 2 | .775 | .379 |
| Profile 1 v. 3 | 11.735 | .001 | Profile 1 v. 4 | 8.561 | .003 |
| Profile 1 v. 5 | 5.414 | .020 | Profile 2 v. 3 | 33.011 | <.001 |
| Profile 2 v. 4 | 20.576 | <.001 | Profile 2 v. 5 | 13.759 | <.001 |
| Profile 3 v. 4 | .002 | .963 | Profile 3 v. 5 | .437 | .509 |
| Profile 4 v. 5 | .274 | .601 |  |  |  |
| **ASI Physical Concerns** | **M** | **SE** |  | **M** | **SE** |
| Profile 1 | 4.352 | .495 | Profile 2 | 4.444 | .201 |
| Profile 3 | 7.857 | .421 | Profile 4 | 7.387 | .647 |
| Profile 5 | **9.728** | .660 |  |  |  |
|  | **Chi-square** | **p-value** |  | **Chi-square** | **p-value** |
| **Overall Test** | 108.039 | <.001 | Profile 1 v. 2 | .030 | .863 |
| Profile 1 v. 3 | 27.613 | <.001 | Profile 1 v. 4 | 13.735 | <.001 |
| Profile 1 v. 5 | 42.384 | <.001 | Profile 2 v. 3 | 47.733 | <.001 |
| Profile 2 v. 4 | 18.908 | <.001 | Profile 2 v. 5 | 58.961 | <.001 |
| Profile 3 v. 4 | .356 | .551 | Profile 3 v. 5 | 5.770 | .016 |
| Profile 4 v. 5 | 6.242 | .012 |  |  |  |
| **ASI Cognitive Concern** | **M** | **SE** |  | **M** | **SE** |
| Profile 1 | 4.896 | .504 | Profile 2 | 4.259 | .207 |
| Profile 3 | 8.124 | .461 | Profile 4 | 8.666 | .663 |
| Profile 5 | 10.463 | .725 |  |  |  |
|  | **Chi-square** | **p-value** |  | **Chi-square** | **p-value** |
| **Overall Test** | 134.337 | <.001 | Profile 1 v. 2 | 1.355 | .244 |
| Profile 1 v. 3 | 21.174 | <.001 | Profile 1 v. 4 | 20.252 | <.001 |
| Profile 1 v. 5 | 39.523 | <.001 | Profile 2 v. 3 | 52.281 | <.001 |
| Profile 2 v. 4 | 40.263 | <.001 | Profile 2 v. 5 | 67.764 | <.001 |
| Profile 3 v. 4 | .431 | .512 | Profile 3 v. 5 | 7.405 | .007 |
| Profile 4 v. 5 | 3.230 | .072 |  |  |  |
| **ASI Social Concerns** | **M** | **SE** |  | **M** | **SE** |
| Profile 1 | 9.123 | .559 | Profile 2 | 7.813 | .225 |
| Profile 3 | 12.420 | .419 | Profile 4 | 11.566 | .613 |
| Profile 5 | 11.860 | .635 |  |  |  |
|  | **Chi-square** | **p-value** |  | **Chi-square** | **p-value** |
| **Overall Test** | 119.871 | <.001 | Profile 1 v. 2 | 4.685 | .030 |
| Profile 1 v. 3 | 21.178 | <.001 | Profile 1 v. 4 | 8.559 | .003 |
| Profile 1 v. 5 | 10.388 | .001 | Profile 2 v. 3 | 83.722 | <.001 |
| Profile 2 v. 4 | 33.088 | <.001 | Profile 2 v. 5 | 36.117 | <.001 |
| Profile 3 v. 4 | 1.28 | .260 | Profile 3 v. 5 | .542 | .462 |
| Profile 4 v. 5 | .107 | .744 |  |  |  |

*Note.* ASI = Anxiety Sensitivity Index. GAD7 = Generalized Anxiety Disorder – 7. DTS = Distress Tolerance Scale.
